# Gamma knife radiosurgery for cerebral cavernous malformations: biologically effective dose predicts therapeutic outcomes

**DOI:** 10.3389/fneur.2025.1691504

**Published:** 2025-11-17

**Authors:** Xiaoman Shi, Hao Deng, Yang Wu, Yuan Gao, Xinyuejia Huang, Senlin Yin, Wei Wang

**Affiliations:** Department of Neurosurgery, West China Hospital, Sichuan University, Chengdu, China

**Keywords:** cerebral cavernous malformation, gamma knife radiosurgery, biologically effective dose, hemorrhage, a retrospective study

## Abstract

**Background:**

Gamma knife radiosurgery (GKRS) is an established option for cerebral cavernous malformations (CCMs) when microsurgical resection is not feasible. Lesion location strongly influences treatment strategy. The biologically effective dose (BED), introduced by J. F. Fowler, has been widely discussed in radiobiology but not evaluated in CCMs.

**Methods:**

A retrospective cohort study was conducted on 107 patients with 123 CCMs treated by GKRS at West China Hospital between June 2020 and December 2022. Post-GKRS hemorrhage was defined as symptomatic bleeding. The annual hemorrhage rate (AHR) quantified bleeding risk, and effective volumetric control was defined as ≥ 20% volume reduction. Clinical outcomes were categorized as improved, stable or worsened.

**Results:**

The mean age was 41 years, and 59.8% were female. Pre-GKRS hemorrhage was most frequent in brainstem (78.6%) and basal ganglia/thalamic lesions (73.3%). During follow-up, 13 patients (10.6%) experienced hemorrhage and AHR decreased from 13.6 to 4.3% per 100 lesion-years (IRR = 0.314; *p* < 0.001). BED was an independent protective factor against postoperative hemorrhage (HR = 0.964, *p* = 0.044) and significantly associated with volumetric and clinical control.

**Conclusion:**

GKRS significantly reduced hemorrhage risk and promoted lesion regression in CCMs. BED was identified as a strong independent predictor of hemorrhage control, volume response and clinical outcomes, outperforming conventional dose metrics. These findings suggest that BED may guide personalized radiosurgical dose optimization for CCMs.

## 1 Introduction

Cerebral cavernous malformations (CCMs) are increasingly identified through brain Magnetic Resonance Imaging (MRI), with an estimated prevalence of 0.2–0.5% (1). Symptomatic CCMs commonly present with hemorrhage, focal neurological deficits, seizures (2, 3) and headaches (4, 5). Previous studies have underscored the importance of lesion location—particularly in the brainstem, basal ganglia, and the thalamus—as a major determinant of both natural hemorrhage risk and surgical accessibility (6–9). Indeed, lesion location remains a pivotal factor in determining the optimal treatment strategy for patients with CCMs.

Over the past two decades, gamma knife radiosurgery (GKRS) has emerged as an effective treatment option for deep-seated or surgically high-risk lesions, despite persistent concerns regarding posttreatment neurological deficits and rebleeding (10–18).

In 1989, the concept of biologically effective dose (BED) was first introduced in radiobiology to describe cell survival, integrating the physical dose – a traditional parameter in GKRS treatment planning – with treatment duration to account for DNA repair during radiation exposure (19). BED has since been considered as a promising predictor for radiosurgical efficacy, as it captures the biological impact on both the target lesion and surrounding healthy tissues (20). Numerous studies have applied the BED framework in the treatment of arteriovenous malformations (AVMs), trigeminal neuralgia, pituitary adenomas, vestibular schwannomas, and meningiomas, yielding clinically significant results (21–27). To date, however, no studies have reported the application of BED in CCMs.

In this study, we systematically analyzed clinical and radiological outcomes of CCMs treated with GKRS across various anatomical locations in a southwestern Chinese population. The primary objective was to investigate potential differences in preoperative characteristics, treatment parameters, and postoperative outcomes among lesions in distinct regions. Specifically, we compared pre- and post-GKRS hemorrhage risk, baseline lesion volume, marginal prescription dose (MPD), dose rate, and BED, to determine their associations with hemorrhage risk reduction, volumetric changes, and symptom control across anatomical subgroups. Our findings provide the first clinical evidence supporting BED as a biologically relevant predictor in CCM radiosurgery, potentially guiding personalized dose optimization.

## 2 Materials and methods

### 2.1 Patient selection

We retrospectively reviewed 107 patients (123 lesions) who underwent GKRS for CCMs at West China Hospital during June 2020 and December 2022. These cases were identified from a cohort of 892 patients diagnosed with CCMs. Inclusion criteria were as follows: a. Diagnosis of CCM confirmed by MRI; b. GKRS performed at the Gamma Knife Center of West China Hospital; c. Initial GKRS as the primary intervention; d. A minimum of 2 years of clinical follow-up; e. Availability of complete medical records and comprehensive follow-up data; f. At least one post-GKRS MRI scan during follow-up. Exclusion criteria included: a. Prior surgical intervention for CCMs; b. Initial GKRS performed at an external institution; c. Loss of follow-up or insufficient data for analysis. Familial CCM was suspected in patients with multiple lesions and/or a positive family history. Genetic testing for CCM-related genes (KRIT1, CCM2, and PDCD10) was performed in selected cases according to institutional policy. Patients with confirmed familial CCM were not excluded from the analysis but were considered in the interpretation of results. The institutional review board at West China Hospital of Sichuan University approved this study (Approval No. 2023–1,534). Because of its retrospective nature, informed consent was not required.

### 2.2 Baseline and follow-up data

Baseline characteristics, dosimetric parameters, and radiographic data were collected for all patients. Clinical outcomes were assessed over a minimum follow-up period of 2 years after GKRS. Each treated lesion was analyzed as an independent observation. The primary outcome was the post-GKRS hemorrhage, expressed as the annual hemorrhage rate (AHR), calculated as the total number of hemorrhagic events by the cumulative follow-up time per lesion. Follow-up time was defined from the date of GKRS to the last clinical evaluation, death, or any subsequent CCM-related surgery. For clinical relevance and consistency with prior studies (11, 28), the pretreatment observation period was defined as the interval from the initial imaging diagnosis or the first hemorrhagic event to the date of GKRS. To avoid overestimation pf the pre-GKRS hemorrhage rate, hemorrhages occurring at initial presentation were excluded from the analysis (5, 29, 30). Thus, the pre-GKRS AHR was defined as the total number of bleeding events- excluding diagnostic bleeding- divided by the cumulative pretreatment duration (in years). The post-GKRS AHR was defined as the total number of post-treatment hemorrhagic events divided by the sum of lesion-specific follow-up years after GKRS.

Secondary outcomes included changes in lesion volume and neurological status. A volumetric reduction of ≥ 20% compared with baseline was considered volumetric control, while clinical control was defined as stabilization or improvement of symptoms based on patients’ subjective reports.

### 2.3 Radiosurgical technique

All treatments were performed using the Leksell Gamma Knife ICON system (Elekta AB, Stockholm, Sweden) between June 2020 and December 2022. After fixation of a stereotactic head frame under local anesthesia, high-resolution MRI, including 1-mm T1- and T2- weighted sequences, was obtained to precisely localize and characterize CCMs. Treatment planning was collaboratively performed by neurosurgeons and radiologists using these images. After confirmation of target coordinates, the patient was positioned within the Gamma Knife unit, and radiosurgery was delivered with rigid head fixation to ensure precision throughout the procedure.

### 2.4 Biological effective dose calculation

The BED for CCMs was calculated based on the simplified model proposed by Jones et al. (20, 31), which facilitates optimization and evaluation of treatment time in GKRS. The model was derived from the Pop et al. study on sublethal damage repair in the rat spinal cord, which follows a biphasic repair process characterized by an *α*/*β* ratio of 2.47 Gy, repair half-times of 0.19 h and 2.16 h, and a repair coefficient (c) of 0.98, yielding *μ*₁ = 3.65 and μ₂ = 0.32. The BED was expressed as Gy_2.47,_ consistent with prior reports involving other intracranial benign conditions (32, 33). The entire treatment time was defined as the sum of the beam-on time and the between-shot intervals, with the latter calculated as 5 min × (n – 1), where n represents the number of shots. BED values were computed using the equation.

### 2.5 Statistical analysis

Statistical analyses were performed using SPSS Statistics version 25 (IBM Corp.) and R software. Descriptive statistics were used to summarize patient and treatment characteristics. Predictors of post-GKRS outcomes including hemorrhage, volumetric control, and clinical status, were evaluated using Cox proportional hazards regression. Variables with *p* < 0.10 in univariate analysis, along with clinically relevant factors (lesion location, volume and hemorrhage history), were entered into the multivariate Cox model to identify independent predictors. Collinear variables were analyzed in separate models to avoid confounding. Results are reported as hazard ratios (HRs) with 95% confidence intervals (CIs). Continuous dosimetric variables were dichotomized according to optimal cut-off values determined by the Youden index from ROC curve analysis for each outcome (see Supplementary Table S1). As no external control group was available, each lesion served as its own control for comparison of pre- and post-GKRS hemorrhage rates. Changes in AHR were analyzed using Poisson regression, with the logarithm of follow-up years as an offset to adjusting for exposure time differences. All analyses were two-tailed and *p* < 0.05 was considered statistically significant.

## 3 Results

### 3.1 Baseline characteristics and GKSR treatment parameters

A total of 107 patients (123 lesions) were included, with a mean age of 41 years (range, 8–73 years) and a female predominance (59.8%). Six patients harbored multiple lesions; two tested positive for CCM gene mutations, one tested negative, and three did not undergo genetic testing. Of all cases, 67 (54.5%) were located in the supratentorial lobar region, 28 (22.8%) in the brainstem, 13(10.6%) in the cerebellum and 15 (12.2%) in the basal ganglia/thalamus. The mean interval between diagnosis and GKRS was 9.3 months (range, 0–121.8 months), and the mean follow-up duration was 38.9 months. The most common presenting symptoms were hemorrhage (49.6%) and headache (46.7%). Baseline demographics and clinical characteristics stratified by lesion location are summarized in Table 1. Patient groups were comparable in mean age, sex distribution, and pretreatment observation period (all *p* > 0.05). In contrast, treatment- and follow-up-related parameters —including dose rate, MPD, prescription isodose line, and total treatment time—differed significantly among locations. A significant difference was also observed in the initial clinical presentation (*p* **<** 0.001), whereas post-GKRS symptomatic outcomes were comparable (*p* = 0.184).

**Table 1:** Baseline characteristics of 107 patients with CCMs collected stratified by anatomical location.

Variable	Overall (*n* = 107)	Supratentorial lobar area (*n* = 59)	Brainstem (*n* = 28)	Cerebellum (*n* = 12)	Basal ganglia/Thalamus (*n* = 15)	*p* value
Demographics (*n*, %)						
No. of lesions	123 (100%)	67 (54.5%)	28 (22.8%)	13 (10.6%)	15 (12.2%)	–
Patients	107 (100%)	59 (55.1%)	28 (26.2%)	12 (11.2%)	15 (14.0%)	–
Age at GKRS, years (mean ± SD)	41.0 ± 1.4	40.4 ± 1.9	37.8 ± 2.5	51.6 ± 5.05	40.4 ± 3.5	0.077
Sex, *n* (%)						0.704
Female	64 (59.8%)	31 (52.5%)	17 (60.7%)	6 (50%)	10 (66.7%)	–
Male	43 (40.2%)	28 (47.5%)	11 (39.3%)	6 (50%)	5 (33.3%)	–
Treatment parameters (mean ± SD)						
Observation period (mo)	9.3 ± 1.9	10.5 ± 3.3	9.2 ± 2.3	10.8 ± 3.9	4.3 ± 1.2	0.348
Follow-up (mo)	38.9 ± 1.5	36.4 ± 1.1	38.0 ± 5.9	37.9 ± 2.7	43.8 ± 4.2	0.014*
Radiologic follow-up (mo)	21.8 ± 1.9	18.7 ± 1.5	22.2 ± 6.9	19.9 ± 3.5	24.0 ± 5.1	0.287
Dose rate (Gy/min)	2.588 ± 0.027	2.574 ± 0.038	2.569 ± 0.050	2.555 ± 0.107	2.714 ± 0.060	0.046*
Number of isocenters mean (range)	3 (2 ~ 6)	3 (2 ~ 6)	3.5 (2 ~ 5)	5 (2.5 ~ 6)	2 (1 ~ 6)	0.429
MPD (Gy)	13.21 ± 0.16	13.61 ± 0.15	11.79 ± 0.46	14.08 ± 0.31	13.33 ± 0.21	0.001*
Prescription isodose line	0.519 ± 0.004	0.517 ± 0.006	0.533 ± 0.008	0.515 ± 0.007	0.503 ± 0.003	0.040*
Treatment time (min)	30.4 ± 1.4	32.5 ± 2.0	23.9 ± 2.1	38.5 ± 4.1	26.1 ± 4.2	0.003*
BED (Gy_2.47_)	67.790 ± 0.966	68.786 ± 1.324	67.015 ± 2.658	64.709 ± 1.298	67.457 ± 1.446	0.378
Clinical presentation before GKRS (*n*, %)						< 0.001*
Hemorrhage	61 (49.59%)	23 (34.33%)	22 (78.57%)	5 (38.46%)	11 (73.33%)	–
Paresthesia	10 (9.30%)	3 (5.08%)	6 (21.43%)	1 (8.33%)	1 (6.67%)	–
Paresis	15 (14.02%)	4 (6.78%)	7 (25.00%)	4 (33.33%)	2 (13.33%)	–
Cranial nerve deficits	19 (17.76%)	2 (3.39%)	17 (60.71%)	1 (8.33%)	1 (6.67%)	–
Headache	50 (46.70%)	27 (45.76%)	15 (53.57%)	3 (25.00%)	8 (53.33%)	–
Loss of consciousness	14 (13.08%)	11 (18.64%)	1 (3.57%)	1 (8.33%)	1 (6.67%)	–
Epilepsy	12 (11.20%)	10 (16.95%)	1 (3.57%)	0	1 (6.67%)	–
Asymptomatic	20 (18.69%)	12 (20.34%)	4 (14.29%)	2 (16.67%)	2 (13.33%)	–
Clinical outcomes (*n*, %)						0.184
Improved	56 (52.3%)	35 (59.3%)	14 (50.0%)	5 (41.7%)	6 (40.0%)	–
Stable	40 (37.4%)	22 (37.3%)	8 (28.6%)	5 (41.7%)	7 (46.7%)	–
New or worsening neurological deficits	11 (10.3%)	2 (3.4%)	6 (21.4%)	2 (16.7%)	2 (13.3%)	–
Volumetric analysis (mean ± SD)						
Pre-GKRS volume (cm^3^)	0.628 ± 0.069	0.698 ± 0.097	0.431 ± 0.122	0.445 ± 0.091	0.845 ± 0.261	0.150
Final follow-up volume (cm^3^)	0.331 ± 0.046	0.341 ± 0.066	0.322 ± 0.104	0.383 ± 0.131	0.262 ± 0.087	0.570
Volume change (*n*, %)						0.106
Enlargement	18 (14.6%)	6 (9.0%)	8 (28.6%)	2 (15.4%)	2 (13.3%)	–
Reduction	105 (85.4%)	61 (91.0%)	20 (71.4%)	11 (84.6%)	13 (86.7%)	–
Effective volumetric control	91 (74.0%)	53 (77.9%)	16 (57.1%)	10 (76.9%)	12 (80.0%)	0.136
Hemorrhage and AHR (*n*, %)						
Hemorrhage after GKRS						
Hemorrhage within 2 years	7 (5.69%)	1 (1.49%)	5 (17.85%)	1 (7.69%)	0	NA
Hemorrhage beyond 2 years	13 (10.57%)	1 (1.49%)	9 (32.14%)	1 (7.69%)	2 (13.33%)	NA
AHR, per 100 lesion-years						
Pre-GKRS AHR	13.6 (13/95.7)	5.1 (3/58.7)	35.0 (7/20)	8.6 (1/11.47)	37.0 (2/5.4)	0.006*
AHR within 2 years	3.3 (8/246)	0.8 (1/134)	10.7 (6/56)	3.9 (1/26)	0	0.220
AHR beyond 2 years	5.9 (9/152.7)	0	13.7 (6/43.8)	0	12.1 (3/24.7)	< 0.001*
Post-GKRS AHR	4.3 (17/398.7)	0.5 (1/203.1)	12.0 (12/99.8)	2.4 (1/41.1)	5.5 (3/54.7)	0.004*

CCM, cerebral cavernous malformation; GKRS, Gamma Knife radiosurgery; MPD, marginal prescription dose; BED, biologically effective dose; AHR, annual hemorrhage rate; mo, months; SD, Standard deviation. Continuous variables are presented as mean ± SD; categorical variables as number (percentage). AHR is expressed per 100 lesion-years. *p < 0.05 was considered statistically significant. *p* values were calculated using one-way ANOVA for continuous variables and χ^2^ tests for categorical variables.

Lesion volumes at baseline (*p* = 0.15), at last follow-up (*p* = 0.57), and the magnitude of volume change (*p* = 0.106) showed no significant differences between groups. However, both the pre- and post-GKRS AHRs varied significantly among anatomical groups (*p* = 0.006 and *p* = 0.004, respectively), with the brainstem and basal ganglia/thalamus groups exhibiting higher rates in both periods.

### 3.2 Hemorrhage

GKRS significantly reduced hemorrhage risk and BED emerged as an independent predictor. Before GKRS, 61 lesions experienced 74 hemorrhagic events, including 13 with multiple bleeds. The pre-GKRS AHR was 77.30 per 100 CM–years, and after excluding diagnostic hemorrhages, the adjusted pre-GKRS AHR was 13.58 (13 events / 95.7 lesion–years). Following GKRS, during 398.67 lesion–years of follow-up, 17 hemorrhages occurred in 13 lesions, yielding a post-GKRS AHR of 4.26.

**Figure 1:**
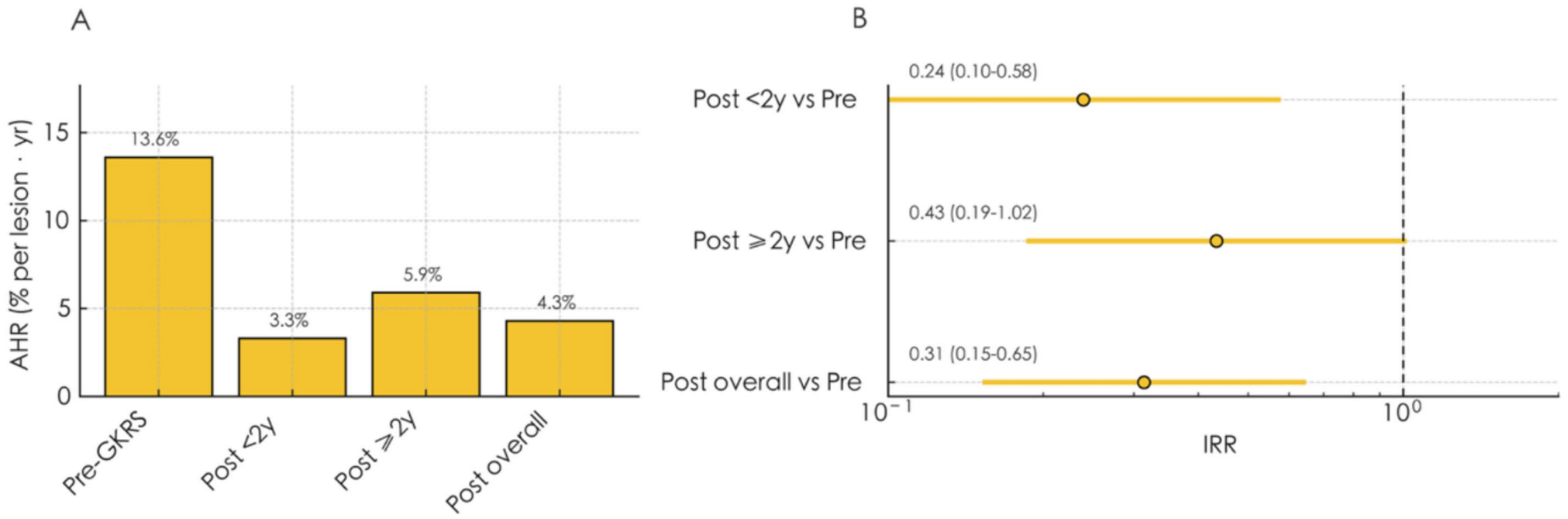
Annual hemorrhage rate and Poisson regression analysis of post-GKRS hemorrhage. **(A)** Comparison of AHRs before GKRS, within 2 years after GKRS (“Early”), beyond 2 years after GKRS (“Late”), and overall post-treatment. **(B)** Incidence rate ratios (IRRs) derived from Poisson regression comparing post-GKRS intervals with the pre-GKRS period. “Early (< 2 years)” and “Late (≥ 2 years)” refer to hemorrhagic events occurring within or beyond 2 years after GKRS, respectively. AHR, Annual hemorrhage rate; GKRS, Gamma knife radiosurgery.

When stratified by follow-up duration, 8 hemorrhages occurred within 2 years (AHR = 3.25; 8 events / 246 lesion–years), and 9 beyond 2 years (AHR = 5.90; 9 events / 152.67 lesion–years), showing no significant difference between intervals (IRR = 1.81; 95% CI 0.70–4.70; *p* = 0.22). Most post-treatment hemorrhages (9/13 lesions) occurred in the brainstem. As illustrated in Figure 1, AHR declined substantially after GKRS, with Poisson regression confirming a 68% reduction in annual bleeding risk (IRR = 0.32; 95% CI, 0.19–0.54; *p* < 0.001).

Univariable and multivariable Cox regression results for predictors of post-GKRS hemorrhage are presented in Table 2. In univariable analysis, MPD, BED, lesion location and BED > 54.224 Gy_2.47_ were significant predictors. Due to multicollinearity, separate multivariable models were built. In the BED model, continuous BED remained a protective factor (aHR = 0.964; 95% CI, 0.93–0.999; *p* = 0.044). Compared with brainstem lesions, supratentorial lobar lesions had significantly lower post-GKRS hemorrhage risk (aHR = 0.083; 95% CI, 0.015–0.445; *p* = 0.004), while cerebellar and basal ganglia/thalamic lesions did not differ significantly. In an alternative model substituting MPD, only supratentorial location remained significant (aHR = 0.10; 95% CI, 0.017–0.572; *p* = 0.01).

**Table 2:** Univariate and multivariate Cox regression analysis identifying of predictors of post-GKRS hemorrhage in all CCMs.

Variables	Univariable		Multivariable (MPD)		Multivariable (BED)	
	HR (95%CI)	*p* value	HR (95%CI)	*p* value	HR (95%CI)	*p* value
Demographics						
Age	0.985 (0.951, 1.020)	0.405			–	–
Sex	0.679 (0.222, 2.077)	0.497			–	–
Observation period (mo)_	0.982 (0.933, 1.035)	0.502			–	–
Radiosurgical parameters					–	–
Dose rate (Gy/min)	0.416 (0.122, 1.412)	0.106			–	–
Number of isocenters	0.956 (0.772, 1.183)	0.679			–	–
MPD (Gy)	0.751 (0.610, 0.925)	0.007*	0.898(0.7, 1.15)	0.396	–	–
Prescription isodose line	0.079 (0.000, 64197.048)	0.715			–	–
BED (Gy_2.47_)	0.939 (0.897, 0.984)	0.008*			0.964 (0.93,0.999)	0.044*
Pre-GKRS volume (cm^3^)	0.354 (0.085, 1.483)	0.155	0.726 (0.289,1.82)	0.495	0.637 (0.246, 1.65)	0.353
Pre-GKRS hemorrhage (yes, n)	0.419 (0.116, 1.361)	0.148	1.22 (0.376, 3.95)	0.742		
Location		0.012*				
Supratentorial lobar area vs. Brainstem	0.042 (0.005, 0.330)	0.003	0.1 (0.017, 0.572)	0.01*	0.083 (0.015, 0.445)	0.004*
Cerebellum vs. Brainstem	0.349 (0.075, 1.622)	0.179	0.566 (0.09, 3.55)	0.543	0.402 (0.072, 2.24)	0.298
Basal ganglia/Thalamus vs. Brainstem	0.218 (0.028, 1.724)	0.149	0.609 (0.141, 2.63)	0.507	0.597 (0.147, 2.43)	0.471

HR, hazard ratio; CI, confidence interval; BED, biologically effective dose; MPD, marginal prescription dose; GKRS, gamma knife radiosurgery; CCMs, cerebral cavernous malformations. Because of multicollinearity among dose-related parameters, two multivariate models were developed—one with MPD and another with BED—to avoid redundancy. *p < 0.05 was considered statistically significant.

### 3.3 Radiological outcomes

**Figure 2:**
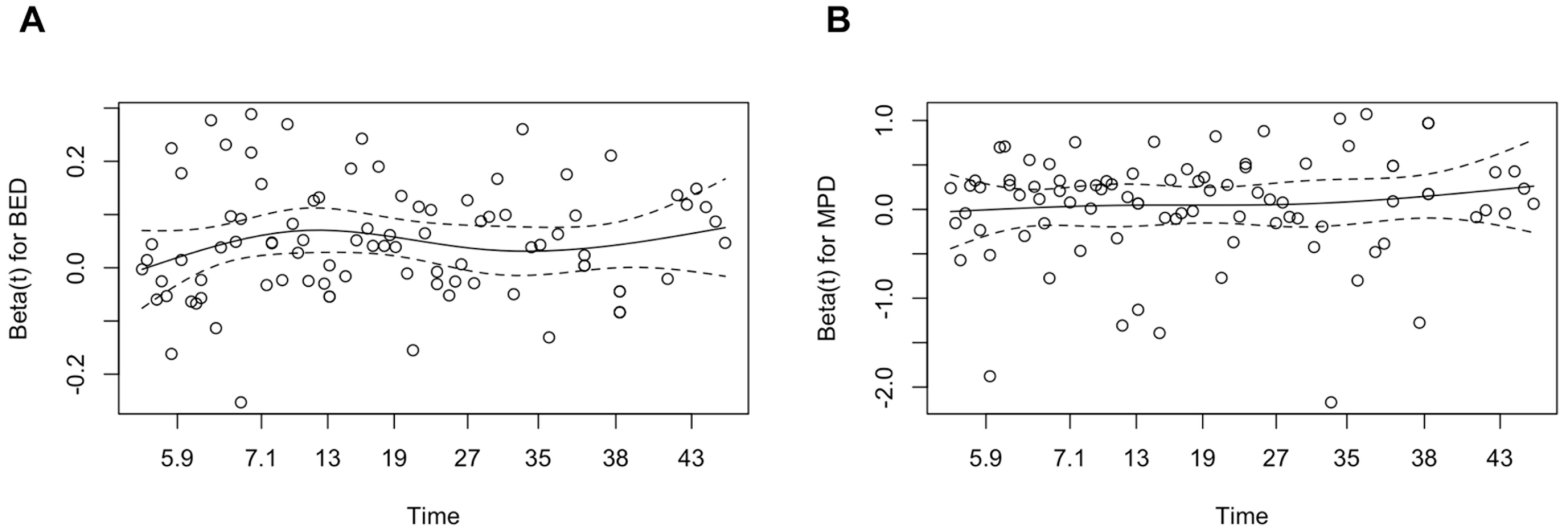
Schoenfeld residual plot for BED **(A)** and MPD **(B)** in the volumetric control model. Schoenfeld residual plots for BED **(A)** and MPD **(B)** against follow-up time, used to assess the proportional hazards assumption in the Cox model for volumetric control. The solid lines represent LOWSS smooth fits, and the dashed lines indicate the zero reference. The residuals are randomly distributed around zero without systematic trends, suggesting that the proportional hazards assumption holds for both BED and MPD. BED, biologically effective dose; MPD, margin prescription dose; LOWESS, Locally weighted scatterplot smoothing.

Higher BED and MPD were independently predicted volumetric control. Among123 lesions with radiological follow-up, 105 (85.4%) decreased in volume and 18 (14.6%) increased. Volumetric control (≥ 20% reduction) was achieved in 74% of lesions. Predictors identified in univariable and multivariable Cox regression are summarized in Table 3. In both models – Model 1 (BED included) and Model 2 (MPD included) –the primary covariate remained significant after adjustment for lesion volume, location and hemorrhage history (all *p* < 0.05). The proportional hazards assumption was confirmed (BED: *p* = 0.53; MPD: *p* = 0.26; global test: *p* = 0.52; Figure 2). MPD was significance was retained when continuous but lost after dichotomization (see Supplementary Table S1).

**Table 3:** Univariate and multivariate Cox regression analysis identifying predictors of post-GKRS volumetric control in All CCMs.

Variables	Univariable		Multivariable (MPD)		Multivariable (BED)	
	HR (95% CI)	*p* value	HR (95% CI)	*p* value	HR (95% CI)	*p* value
Demographics						
Age	1.003 (0.988, 1.017)	0.718	–	–	–	–
Sex	0.978 (0.645, 1.484)	0.918	–	–	–	–
Observation period (mo)	0.996 (0.983, 1.009)	0.527	–	–	–	–
Radiosurgical parameters						
Dose rate (Gy/min)	1.367 (0.736, 2.540)	0.057	–	–	–	–
Number of isocenters	1.032 (0.955, 1.116)	0.420	–	–	–	–
MPD (Gy)	1.298 (1.130, 1.492)	0.016*	1.170 (1.010, 1.340)	0.030*	–	–
Prescription isodose line	0.215 (0.004, 11.830)	0.452	–	–	–	–
BED (Gy_2.47_)	1.051 (1.029, 1.074)	< 0.001*	–	–	1.050 (1.030, 1.080)	<0.001*
Pre-GKRS volume (cm^3^)	1.161 (0.910, 1.481)	0.230	1.050 (0.793, 1.380)	0.745	1.100 (0.820, 1.470)	0.534
Pre-GKRS hemorrhage (yes, n)	0.934 (0.615, 1.420)	0.751	1.330 (0.826, 2.130)	0.242	1.080 (0.664, 1.750)	0.760
Location	0.864 (0.699, 1.068)	0.177				
Supratentorial lobar area vs. Brainstem	–	–	1.27 (0.675, 2.41)	0.745	1.57 (0.869, 2.84)	0.135
Cerebellum vs. Brainstem	–	–	1.01 (0.404, 2.52)	0.984	1.56 (0.654, 3.71)	0.317
Basal ganglia/Thalamus vs. Brainstem	–	–	0.726(0.334, 1.58)	0.419	0.857 (0.397, 1.85)	0.695

HR, hazard ratio; CI, confidence interval; BED, biologically effective dose; MPD, marginal prescription dose; GKRS, gamma knife radiosurgery; CCMs, cerebral cavernous malformations. Because of multicollinearity among dose-related parameters, two multivariate models were developed—one with MPD and another with BED—to avoid redundancy. *p < 0.05 was considered statistically significant.

**Figure 3:**
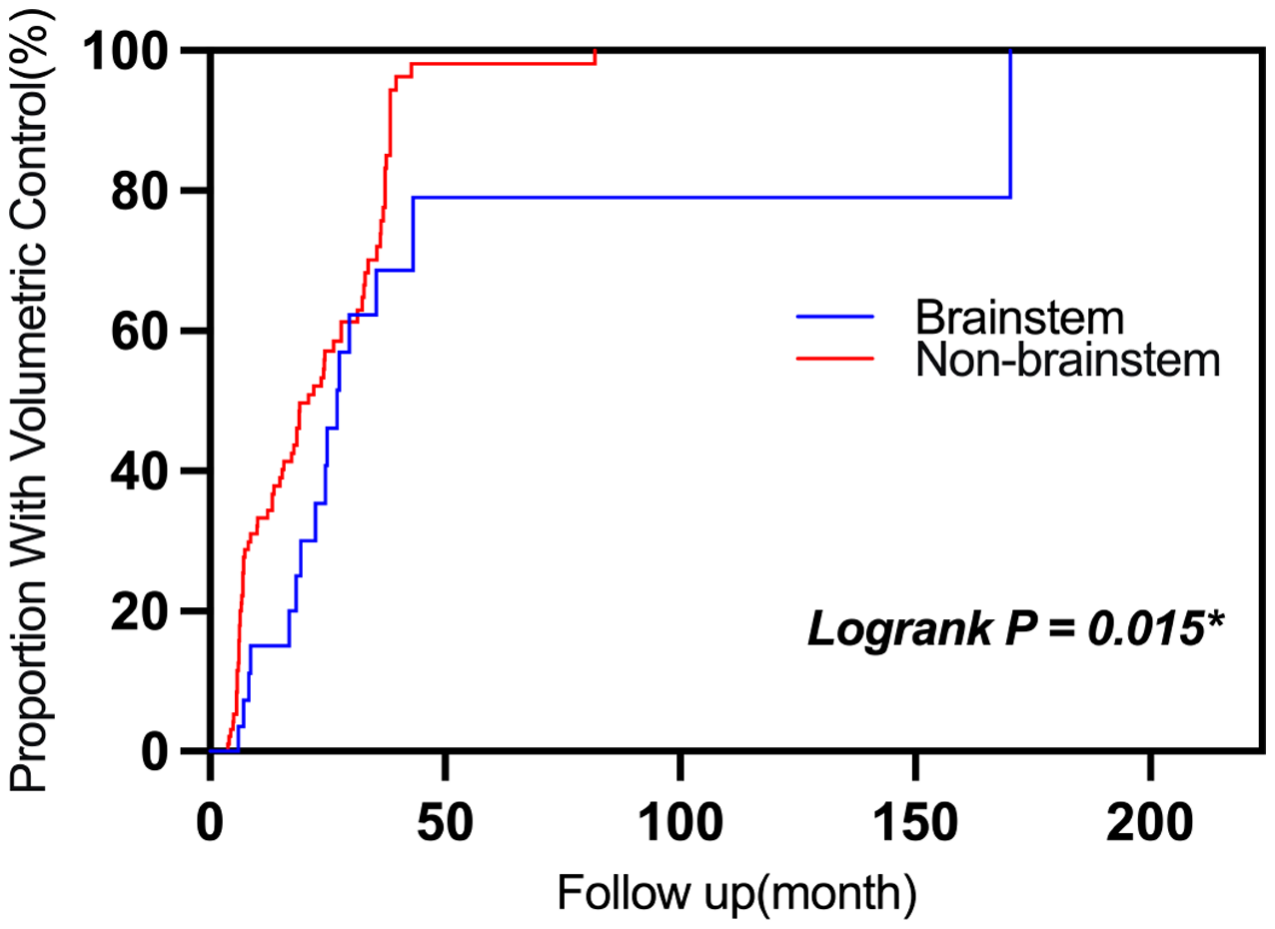
Cumulative hazard curves for achieving volumetric control after GKRS in CCMs. Cumulative hazard curves illustrate the time-dependent probability of achieving ≥20% lesion volume reduction following GKRS. Lesions located in the brainstem (blue) showed a significantly lower likelihood of volumetric control compared with non-brainstem lesions (red) (*p* = 0.015, log-rank test). AHR, Annual hemorrhage rate; GKRS, Gamma knife radiosurgery.

Lesion location was not a univariable predictor (*p* = 0.177), but brainstem lesions required a longer time to achieve volumetric control than non-brainstem ones (*p* = 0.015; Figure 3).

### 3.4 Clinical outcomes

BED was the only consistent predictor of clinical control. Over a mean clinical follow-up of 38.9 months, 56 patients (52.3%) experienced clinically, 40 (37.4%) remained stable, and 11 (10.3%) developed new or progressive neurological deficits, predominantly in brainstem lesions (54.5%). Six patients (5.6%) underwent surgical intervention and no treatment-related deaths occurred.

Results of the Cox regression analysis for clinical outcome predictors are shown in Table 4. In univariate Cox analysis, higher BED (HR = 1.060; 95% CI, 1.038–1.083; *p* < 0.001), greater number of isocenters (HR = 1.007; 95% CI, 1.001–1.144; *p* = 0.048), and higher dose rate (HR = 0.512; 95% CI, 0.341–0.768; *p* = 0.001) were significantly associated with favorable clinical outcome. In the multivariate model included BED, it remained an independent predictor (aHR = 1.07; 95% CI, 1.04–1.09; *p* < 0.001), while lesion volume showed only a marginal trend (aHR = 1.21; 95% CI, 0.97–1.51; *p* = 0.091). No significant associations were observed for age, sex, observation period, MPD, pre-SRS hemorrhage status, or lesion location (all *p* > 0.05). However, significant correlations were observed in high BED, large volume, high MPD and high dose rate (see Supplementary Table S1).

**Table 4:** Univariate and multivariable Cox regression analysis identifying predictors of post-GKRS clinical control in All CCMs.

Variables	Univariable		Multivariable (BED)	
	HR (95% CI)	*p* value	HR (95% CI)	*p* value
Demographics				
Age	0.998 (0.986, 1.011)	0.807	–	–
Sex	1.043 (0.295, 3.685)	0.948	–	–
Observation period (mo)	0.999 (0.989, 1.009)	0.844	–	–
Radiosurgical parameters				
Dose Rate (Gy/min)	1.470 (0.909, 2.378)	0.116	–	–
Number of isocenters	1.007 (1.001, 1.144)	0.048*	–	–
MPD (Gy)	1.149 (0.785,1.683)	0.475	–	–
Prescription isodose line	14.448 (0.358, 583.323)	0.157	–	–
BED (Gy_2.47_)	1.060 (1.038, 1.083)	< 0.001*	1.070 (1.040, 1.090)	< 0.001*
Pre-GKRS volume (cm^3^)	1.059 (0.854, 1.314)	0.600	1.210 (0.970, 1.510)	0.091
Pre-GKRS hemorrhage (yes, n)	0.976 (0.671, 1.420)	0.899	0.855 (0.566, 1.290)	0.457
Location	1.351 (0.824, 2.217)	0.233	–	–
Supratentorial lobar area vs. Brainstem	–	–	1.23 (0.743, 2.050)	0.417
Cerebellum vs. Brainstem	–	–	1.600 (0.756, 3.400)	0.219
Basal ganglia/Thalamus vs. Brainstem	–	–	0.576 (0.279, 1.190)	0.137

HR, hazard ratio; CI, confidence interval; BED, biologically effective dose; MPD, marginal prescription dose; GKRS, gamma knife radiosurgery; CCMs, cerebral cavernous malformations. Because of multicollinearity among the dosimetric parameters (dose rate, number of isocenters, MPD, and BED), only BED was included in the final multivariate model. *p < 0.05 was considered statistically significant.

## 4 Discussion

This retrospective single-center study provided the first clinical evidence that BED served as a biologically relevant predictor of treatment response in CCMs treated with GKRS. Our results demonstrated that a higher BED was independently associated with reduced post-GKRS hemorrhage risk, effective volumetric control, and better clinical outcomes. These findings suggest that incorporating BED into GKRS planning could enhance the biological precision of treatment, complementing traditional physical dose metrics such as MPD and dose rate.

### 4.1 Natural history of cerebral cavernous malformations

Several studies have reported potential risk factors associated with rebleeding in conservatively managed CCMs, including age (34–36), lesion size or diameter (35), the presence of developmental venous anomalies (DVAs) (37, 38), lesion location (7, 39–42), and prior hemorrhage history (37–39, 43, 44). However, many of these associations remain under debate. Due to the heterogeneous distribution of critical neural nuclei and eloquent structures within the brain, CCMs in different anatomical regions present with distinct clinical profiles (39). In our cohort, lesions located in the brainstem and basal ganglia/thalamus were more likely to present with hemorrhage and neurological deficits. AHR serves as a key metric for quantifying bleeding risk before and after treatment, but its calculation has varied substantially across studies– particularly in low-frequency hemorrhagic events and observation period are defined. Common approaches include: A. Considering CMs as congenital lesions, with AHR calculated as the total number of pre-treatment hemorrhages divided by patient age in years (45); B. Excluding the initial diagnostic hemorrhage, where AHR equals the number of subsequent hemorrhages divided by lesion-years from diagnosis (13); C. Including all pre-treatment hemorrhages, divided by lesion-years from diagnosis (16). Among these, excluding the initial diagnostic hemorrhage provides a more accurate reflection of post-diagnosis risk, facilitates consistent comparison with post-treatment hemorrhage rates, and prevents overestimation of baseline hemorrhagic risk. Consistent with this rationale, our study adopted the approach of excluding the initial presenting hemorrhage when calculating AHR. Besides, the presence of familial CCM cases may represent a potential source of bias, as these patients are more prone to recurrent hemorrhage compared with sporadic cases. However, genetic findings were considered during data interpretation, and the limited number of familial cases is unlikely to have significantly influenced the overall results.

### 4.2 Radiosurgical management of cerebral cavernous malformations

CCMs are benign vascular malformations of the central nervous system. Unlike AVMs, they are angiographically occult. A defining pathological feature—the absence of tight junctions between endothelial cells—confers distinct radiobiological behavior. In contrast to AVMs, CCM lumens rarely achieve complete obliteration following irradiation, partly due to the relative paucity of radiation-sensitive endothelial components (46).

Our analysis confirmed that GKRS serves as a significant protective factor against recurrent hemorrhage (IRR = 0.316; 95% CI: 0.185–0.537; *p* < 0.001), corresponding to a 68.4% reduction in annualized hemorrhage risk. Thes findings align with previous studies demonstrating reduced post-GKRS hemorrhage rates (Table 5) (13, 14, 28, 47–54). The difference in AHR beyond 2 years post-GKRS was not statistically significant in our cohort, consistent with one meta-analysis (11) but different from another (55). Collectively, current evidence supports a decline in AHR following radiosurgical intervention.

**Table 5:** Annual hemorrhage rates before and after stereotactic radiosurgery: summary of data extracted from 11 included studies.

Paper number	Pre-SRS AHR	After-SRS within 2 years AHR	After-SRS beyond 2 years AHR	After-SRS AHR
Kefeli et al. (47)	8.6%	1.22%	0.56%	0.87%
Liu et al. (48)	25%	3.92%	1.85%	3.07%
Kida et al. (49)	21.48%	7.4%	2.80%	4.36%
Kim et al. (50)	7.26%	2.63%	0.61%	1.26%
Frischer et al. (51)	33.56%	8.14%	2.37%	4.80%
Park and Hwang (52)	39.57%	8.20%	0%	1.54%
Lee et al. (28)	31.32%	4.29%	3.64%	3.94%
Monaco et al. (53)	32.38%	8.22%	1.37%	3.87%
Choudhri et al. (54)	33.90%	12.32%	0.76%	4.75%
Karaaslan et al. (13)	15.3%	2.6%	1.4%	–
Li et al. (14)	23.6%	9.02%	7.52%	–

SRS, Stereotactic surgery; AHR, Annual hemorrhage rate; dashes (—) indicate variables not included in articles.

The radiobiological mechanism is thought to involve endothelial injury and inflammation, leading to fibrinoid necrosis with or without thrombosis, followed by fibrotic scarring that progressively narrows or occludes the vascular lumen. This process provides a mechanistic basis for the gradual reduction in hemorrhage risk as lesional blood flow becomes remodeled or sealed off (56, 57). In our cohort, hemorrhage rates within 2 years after GKRS and beyond 2 years did not differ significantly, suggesting a sustained treatment effect. This contrasts with the natural history of untreated CCMs, which often exhibits temporal clustering of hemorrhages (34, 43). Therefore, to more convincingly establish the therapeutic effect of radiation, post-treatment hemorrhage data should ideally be compared between radiosurgically treated and conservatively managed cohorts.

Nagy et al. identified younger age, deep lesion location, and multiple pre-treatment hemorrhages as predictors of post-GKRS bleeding (58). In our study, BED and lesion location emerged as stable predictors of hemorrhage risk in both univariable and multivariable Cox analyses. Lesions located in brainstem exhibited the highest risk and those in supratentorial lobar the lowest. Notably, a history of prior hemorrhage did not significantly predict post-tradiosurgical bleeding, possibly because radiation-induced structural remodeling alters the natural risk of bleeding.

Radiologically, 85.4% of lesions decreased in size after GKRS, and 74% achieved volumetric control, consistent with previous reports (16, 55). These findings further support the efficacy of GKRS in promoting CCM regression. Higher BED and MPD (as continuous variables) were independently associated with faster and more likely achievement of volumetric control. The significant correlation of MPD as a continuous variable, but not after dichotomization, suggests a potential dose–response association between MPD and post-GKRS volumetric control. However, categorizing the variable reduced statistical power due to uneven group distribution.

Lesions located in the brainstem required a longer duration to achieve volumetric control, likely due to receiving lower MPD during radiosurgery. Overall, volume changes appear to be primarily driven by radiation-induced pathological remodeling and are closely related to GKRS dosimetric parameters.

### 4.3 Biologically effective dose

The concept of BED and its formulation was first introduced by J. F. Fowler in the British Journal of Radiology in 1989 (19). This metric highlights the impact of treatment time in stereotactic radiosurgery. Our baseline data reveal considerable variation in treatment time, primarily attributable to differences in the Co-60 source dose rate and the degree of automation of the radiosurgical device.

Currently, GKRS treatment planning relies on physical radiation doses parameters- MPD, dose distribution map, dose-volume histogram, and target-specific irradiation dose and duration in GammaPlan- as the clinical gold standard (20). In contrast, BED offers additional insight by incorporating biological effects into the evaluation.

BED has been extensively investigated in central nervous system diseases treated with GKRS, yielding encouraging results and establishing it as a promising metric for radiotherapy planning and outcome assessment. For instance, BED accurately predicts AVM obliteration rates (21, 59, 60). In trigeminal neuralgia, BED—specifically within a range of 1820–1962.5 Gy_2.47_—has emerged as the critical predictor of efficacy, outperforming MPD and achieving an optimal balance between therapeutic effect and toxicity (22, 61, 62). Similarly, BED correlates with biochemical remission in acromegaly, Cushion disease and Hypopituitarism (25, 32, 63–65); shows a significant association with tumor volume reduction in vestibular schwannomas (23, 24, 66, 67); and predicts treatment failure in meningiomas, where MPD alone lacks statistical significance (27, 68).

Despite these broad applications, no published data have yet addressed the role of BED in CCMs. The present study fills this gap by establishing BED as a strong and independent predictor of treatment outcome in patients with CCMs. Given these novel findings, further multicenter studies are warranted to validate the predictive role of BED and to refine its clinical practice.

### 4.4 Limitations

This study has several limitations. First, its monocentric origin may limit the external validity of the findings. As highlighted in the CARE trial, multicentre recruitment would be desirable to better define the burden and clinical characteristics of this rare entity. In addition, the relatively short follow-up period restricts assessment of long-term outcomes. Besides, its retrospective design introduces inherent selection bias, and the absence of a natural-history control group limits causal inference. Treatment parameters (e.g., MPD) were not randomized but chosen at physicians’ discretion, creating confounding by indication and the inclusion of patients with unbled sporadic cavernomas was based on our institutional treatment policy, which considers surgical or radiosurgical intervention in selected cases depending on lesion location, size, and symptomatic presentation. Symptom burden was not quantified using standardized scales such as the modified Rankin Scale, reducing comparability. Patients lost to follow-up were mainly asymptomatic or without recurrent hemorrhage, potentially inflating the observed rebleeding rate, while the extent of loss was not systematically recorded. Given the uncertain onset of GKRS treatment effects in CCMs, the causal strength of our conclusions is limited.

These results should therefore be interpreted with caution, and larger, prospective studies with standardized outcome assessment and extended follow-up are needed to confirm durability and refine dose optimization.

## 5 Conclusion

This study demonstrates that GKRS was associated with a reduced hemorrhage rate. Beyond confirming the protective role of radiosurgery, our analysis identified BED as a powerful and independent predictor of treatment outcomes, surpassing traditional dosimetric parameters such as MPD. The incorporation of BED into treatment planning may allow clinicians to better individualize GKRS strategies, balancing efficacy with safety, and thereby improving long-term patient outcomes. As no prior data have addressed the role of BED in CCMs, our findings establish a new framework for integrating radiobiological principles into clinical decision-making. Prospective multicenter studies are needed to validate these results and to translate BED-guided radiosurgery into routine practice for optimal patient care.

## Data Availability

The raw data supporting the conclusions of this article will be made available by the authors, without undue reservation.
