# Supplementary material for: Gamma knife radiosurgery for cerebral cavernous malformations: biologically effective dose predicts therapeutic outcomes
Source: Front Neurol. 2025 Nov 17;16:1691504. doi: 10.3389/fneur.2025.1691504 (PMC12665552; doi:10.3389/fneur.2025.1691504)
Supplement: Supplementary file 1 [file Table_1.docx]

Supplementary Material

## Supplementary Table

**Supplementary Table S1: Optimal cut-off values of continuous dosimetric variables determined by the Youden index**

| **Outcome** | **Variable** | **Cut-off value** | **Unit** | **Definition of high group** | **Method of determination** | **Univariate**  **HR (95%CI)** | **P value** |
| --- | --- | --- | --- | --- | --- | --- | --- |
| **Post-hemorrhage outcome** | Lesion volume | 0.21 | cm³ | > 0.21 cm³  (“large volume”) | ROC analysis using Youden index | 1.987  (0.64, 6.169) | 0.235 |
|  | MPD | 12.5 | Gy | > 12.5 Gy  (“high MPD”) | ROC analysis using Youden index | 0.387  (0.145, 1.035) | 0.059 |
|  | Dose rate | 2.62 | Gy/min | > 2.62 Gy/min  (“high dose rate”) | ROC analysis using Youden index | 0.376  (0.133, 1.065) | 0.066 |
|  | BED | 54.224 | Gy_2.47_ | > 54.224 Gy_2.47_  (“high BED”) | ROC analysis using Youden index | 0.138  (0.044, 0.436) | 0.001* |
| **Volumetric control outcome** | Lesion volume | 0.386 | cm³ | > 0.386 cm³  (“large volume”) | ROC analysis using Youden index | 0.783  (0.474, 1.293) | 0.510 |
|  | MPD | 11.0 | Gy | > 11 Gy  (“high MPD”) | ROC analysis using Youden index | 0.408  (0.236, 0.705) | 0.201 |
|  | Dose rate | 2.461 | Gy/min | > 2.461 Gy/min  (“high dose rate”) | ROC analysis using Youden index | 1.637  (1.047, 2.559) | 0.055 |
|  | BED | 66.598 | Gy_2.47_ | > 66.598 Gy_2.47_  (“high BED”) | ROC analysis using Youden index | 0.502  (0.327, 0.771) | 0.001* |
| **Clinical control outcome** | Lesion volume | 0.21 | cm³ | > 0.21 cm³  (“large volume”) | ROC analysis using Youden index | 0.995  (0.680, 1.457) | 0.979 |
|  | MPD | 12.5 | Gy | > 12.5 Gy  (“high MPD”) | ROC analysis using Youden index | 1.756  (1.135, 2.716) | 0.012* |
|  | Dose rate | 2.62 | Gy/min | > 2.62 Gy/min  (“high dose rate”) | ROC analysis using Youden index | 0.512  (0.341, 0.768) | 0.001* |
|  | BED | 56.959 | Gy_2.47_ | > 56.959 Gy_2.47_  (“high BED”) | ROC analysis using Youden index | 3.524  (1.754, 7.080) | < 0.001* |

Abbreviations: MPD, marginal prescription dose; BED, biologically effective dose; ROC, receiver operating characteristic; *p < 0.05 was considered statistically significant. Optimal thresholds were determined by maximizing the Youden index (sensitivity + specificity − 1) for predicting each postoperative outcome (hemorrhage, volumetric control, or clinical control).
